# Gene expression profiling of chondrogenic differentiation by dexamethasone-conjugated polyethyleneimine with SOX trio genes in stem cells

**DOI:** 10.1186/s13287-018-0998-7

**Published:** 2018-12-07

**Authors:** Se Won Yi, Hye Jin Kim, Hyun Jyung Oh, Heejun Shin, Jung Sun Lee, Ji Sun Park, Keun-Hong Park

**Affiliations:** 1 Department of Nano-regenerative Medical Engineering, College of Life Science, CHA University, 335, Pangyo-ro, Bundang-gu, Seongnam-si, 134-88 Republic of Korea; 2 Department of Biotechnology, Catholic University 43-1, Yeokgok 2-dong, Wonmi-gu, Bucheon-si, Gyeonggi-do 420-743 Republic of Korea

**Keywords:** hMSC, Dexamethasone, PEI, DI-NP, SOX trio, mRNA profiling

## Abstract

**Background:**

During differentiation of stem cells, it is recognized that molecular mechanisms of transcription factors manage stem cells towards the intended lineage. In this study, using microarray-based technology, gene expression profiling was examined during the process of chondrogenic differentiation of human mesenchymal stem cells (hMSCs). To induce chondrogenic differentiation of hMSCs, the cationic polymer polyethyleneimine (PEI) was coupled with the synthetic glucocorticoid dexamethasone (DEX). DEX/PEI could be polyplexed with anionic plasmid DNAs (pDNAs) harboring the chondrogenesis-inducing factors SOX5, SOX6, and SOX9. These are named differentiation-inducing nanoparticles (DI-NPs).

**Methods:**

A DI-NP system for inducing chondrogenic differentiation was designed and characterized by dynamic light scattering and scanning electron microscopy (SEM). Chondrogenic induction of hMSCs was evaluated using various tools such as reverse-transcription polymerase chain reaction (RT-PCR), Western blotting, confocal fluorescent microscopy, and immunohistochemistry analysis. The gene expression profiling of DI-NP-treated hMSCs was performed by microarray analysis.

**Results:**

The hMSCs were more efficiently transfected with pDNAs using DI-NPs than using PEI. Moreover, microarray analysis demonstrated the gene expression profiling of hMSCs transfected with DI-NPs. Chondrogenic factors including SOX9, collagen type II (COLII), Aggrecan, and cartilage oligometric matrix protein (COMP) were upregulated while osteogenic factors including collagen type I (COLI) was downregulated. Chondrogenesis-induced hMSCs were better differentiated as assessed by RT-PCR, Western blotting analyses, and immunohistochemistry.

**Conclusion:**

DI-NPs are good gene delivery carriers and induce chondrogenic differentiation of hMSCs. Additionally, comprehensive examination of the gene expression was attempted to identify specific genes related to differentiation by microarray analysis.

**Graphical abstract:**

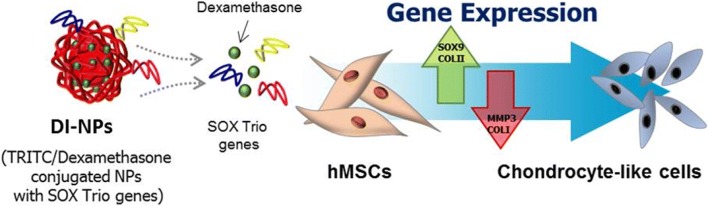

**Electronic supplementary material:**

The online version of this article (10.1186/s13287-018-0998-7) contains supplementary material, which is available to authorized users.

## Background

Gene therapy is a revolutionary way to treat the causes of diseases at the gene level. In addition, it is possible to achieve long-term expression in vivo, and genetic information mutated by genetic recombination can be corrected. Many studies have focused on gene delivery as a promising tool to treat diseases at the genetic level [1, 2]. Genes encoding transcription factors, which regulate signaling pathways related to cell proliferation, growth, differentiation, and death, have been the focus of great interest [3–6]. The internalization of genes encoding transcription factors related to stem cell differentiation has been extensively studied [7–10]. Cells have been transfected with genes encoding specific transcription factors in an attempt to enhance differentiation towards the desired lineage and prevent differentiation towards other lineages [11, 12]. In this regard, we designed and synthesized dexamethasone (DEX)-conjugated polyethyleneimine (PEI), called DEX/PEI, as a gene carrier. DEX is a synthetic glucocorticoid and therefore binds to the glucocorticoid receptor in the cytosol via the ligand-receptor complex [13] after transfection of DEX/PEI. The complex is translocated to the nucleus, resulting in enlargement of the nuclear pore; this is helpful for gene delivery. Furthermore, DEX is already known to be an important factor for mesenchymal stem cells leading to osteoblasts, adipocytes, and chondrocytes [14], and DEX also alleviates inflammation. By conjugating DEX to PEI, the efficiency of gene delivery could be improved, facilitating induction of differentiation with low cytotoxicity and low effects of inflammation when applied to an in-vivo study [15].

Cationic PEI of DEX/PEI could be easily complexed with anionic plasmid DNA (pDNA) harboring three master chondrogenesis factors: SOX5 (specifically L-SOX5), SOX6, and SOX9 [16]. SOX9 is expressed in chondrogenic progenitor cells and mature chondrocytes and induces expression of cartilage-related genes such as collagen type 2 alpha 1 and aggrecan [17]. L-SOX5 and SOX6 are excited at the stage of prechondrocytes in the presence of SOX9 and increase cartilage-specific gene expression together with the existence of SOX9 [18]. These DEX/PEI complexes with pDNA of SOX5, SOX6, and SOX9 are termed differentiation-inducing nanoparticles (DI-NPs), and DI-NPs could effectively induce chondrogenic differentiation of stem cells. Mesenchymal stem cells (MSCs) are one of the broadly used stem cell types for repairing cartilage defects [19–21]. In this study, DI-NPs were applied to MSCs for inducing chondrogenic differentiation. The differentiation of MSCs into chondrocytes has not, however, been overly studied for gene expression profiling using microarray [22]. Through gene expression profiling, it is possible to measure and compare the expression level of mRNAs in differentiation-induced cells and noninduced cells. This allows the identification of genes that are overexpressed or underexpressed in differentiation-induced cells. The expression profile may provide information regarding the level of differentiation of an induced model by a particular tool [23, 24]. Gene expression profiling using microarrays is a major tool for the measurement of levels and patterns of gene expression. It can be used to identify diseases and uncover treatments in clinical medicine. Moreover, analyses of gene expression can be clinically useful for therapeutic application of stem cells toward the treatment of diseases related to cartilage defects.

**Scheme 1:**
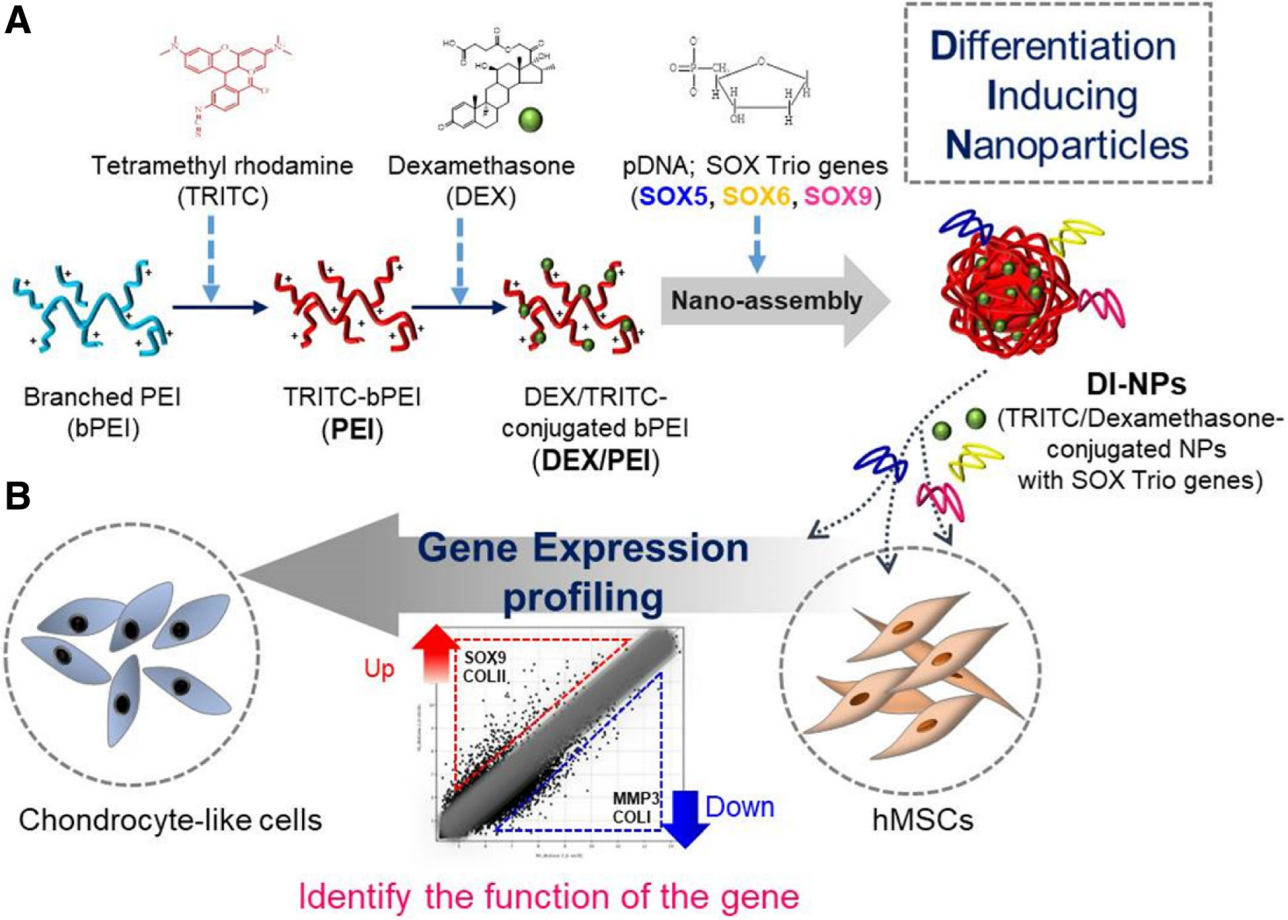
Schematic diagram of the preparation of DI-NPs to induce chondrogenesis of hMSCs. **A** Dexamethasone (DEX)/tetramethylrhodamine (TRITC)-conjugated branched polyethyleneimine (bPEI) (DEX/PEI) is made by the serial conjugation of bPEI and DEX. The DEX/PEI is complexed with SOX trio genes (SOX5, SOX6, and SOX9), called differentiation inducing nanoparticles (DI-NPs). **B** Transfection of DI-NPs induces chondrogenic differentiation of human mesenchymal stem cells (hMSCs). The DI-NPs enter hMSCs via endocytosis. The plasmid DNAs (pDNAs) are released from DI-NPs into the cytosol and the expression levels of specific genes, related to chondrogenesis, are changed (up and down); finally, chondrogenic differentiation of hMSCs is induced

Here, DI-NPs were characterized and evaluated as gene carriers in stem cells. Gene expression profiling was performed using a microarray to evaluate the differentiation of human MSCs (hMSCs) following the delivery of the SOX trio of genes (SOX5, SOX6, and SOX9) [25, 26]. Gene expression was also compared between hMSCs transfected with one gene and those transfected with all three genes. Transfected hMSCs cultured in two- and three-dimensional (3D) systems were further assessed by reverse-transcription polymerase chain reaction (RT-PCR), Western blotting, fluorescence-activated cell sorting (FACS), and immunohistochemistry. The study procedure is illustrated in Scheme 1.

## Methods

### Preparation of nanoparticles (PEI and DEX/PEI)

Tetramethylrhodamine (TRITC; 1 mg; Sigma Aldrich, St. Louis, MO, USA) dissolved in 3 mL dimethyl sulfoxide (DMSO) was mixed with 500 mg branched PEI (bPEI; 25,000 Da; Sigma Aldrich, St. Louis, MO, USA) dissolved in 10 mL distilled water for 12 h at room temperature. The solution was then dialyzed (molecular weight cut-off (MWCO), 1000 Da) for 2 days in the dark; and TRITC-conjugated bPEI (PEI) were synthesized. TRITC/DEX-conjugated bPEI (DEX/PEI) was synthesized via a conventional carbodiimide reaction. The bPEI (250 mg, 10 μmol) was stirred in 10 mL DMSO until completely dissolved. Then, a mixture of DEX (25 mg, 50.76 μmol; Steraloids, Newport, RI, USA), dicyclohexylcarbodiimide (DCC; 16 mg, 77.55 μmol), and n-hydroxysuccinimide (NHS; 16 mg, 139.02 μmol) prepared in 5 mL DMSO was added to the bPEI solution and incubated for 24 h at room temperature. Subsequently, 1 mg TRITC dissolved in 3 mL DMSO was added dropwise to this solution over 12 h. The final solution was dialyzed (MWCO, 1000 Da) against distilled water for 3 days. The final products were obtained by lyophilization.

### Characterization of nanoparticles

First, the size and surface charge of the nanoparticles (NPs) were measured with a Zetasizer Nano ZS apparatus (Malvern, USA). NPs were dispersed with distilled water. Twenty measurements were conducted per sample. The mean diameter and size distribution of NPs was evaluated, and morphology of NPs was visualized by scanning electron microscopy (SEM). A microscope coverglass (18 mm diameter; 0111580) was coated with NPs and dried overnight. The samples were coated with platinum using a PT Sputter (E-1030, Hitachi, Japan) and finally visualized by SEM (S-4700, Hitachi, Japan). The critical aggregation concentrations (CACs) of PEI and DEX/PEI were determined with a fluorescence spectrophotometer (RF-5301PC, Shimadzu, Japan) using Hoechst 33342 as a fluorescent probe. Briefly, a stock solution of Hoechst 33342 (1.4 × 10^–3^ M) was prepared in double-distilled water. Various concentrations of DEX/PEI (2.4 × 10^–4^ to 0.5 mg/mL) were added to this solution. The final concentration of Hoechst 33342 was 7.0 × 10^−4^ M in each case. Fluorescence was measured with excitation and emission wavelengths of 355 and 457 nm, respectively.

### Cell culture

The hMSCs, isolated from the bone marrow of a 20-year-old male, were purchased from Lonza Walkersville Inc. (PT-2501) and cultured in alpha minimum essential medium supplemented with 10% fetal bovine serum and 1% antibiotic-antimycotic solution. For the 3D culture system, the hMSCs were pelleted in a 15-mL conical tube by centrifugation at 1300 rpm for 3 min. The cell pellets were cultured for 3 weeks, changing the medium every 2–3 days, and maintained their ball-like shape.

### Triple gene transfection of hMSCs using trio-coated PEI and DI-NPs

The hMSCs were transfected with pDNAs harboring SOX5, SOX6, and SOX9 using the optimal concentrations of PEI and DEX/PEI (trio-coated PEI and DI-NPs) in serum-free medium for 20 min and incubated for 4 h. Thereafter, SOX5/6/9 protein expression was evaluated by FACS, Western blotting, and fluorescence confocal microscopy (LSM 880 META, Zeiss).

### Evaluation of the cytotoxicity of trio-coated PEI and DI-NPs

Cytotoxicity was evaluated with a cell counting kit (CCK)8 kit and a Live/Dead kit (Invitrogen, Carlsbad, CA, USA). A total of 2 × 10^5^ hMSCs, treated with trio-coated PEI or DI-NPs, were treated with CCK8 solution for 2 h at 37 °C. The optical density at 450 nm was measured using a spectrophotometer. The hMSCs, treated with trio-coated PEI or DI-NPs, were incubated in a solution containing 2 μM calcein AM (to stain live cells) and 4 μM EthD-1 (to stain dead cells) for the Live/Dead assay, and visualized by fluorescence microscopy (EVOS Fl). The hMSCs were treated with 1, 3, 5, 7, and 10 μg DI-NPs for 4 h. The state of the cells treated with 1 μg DI-NPs was confirmed by FACS.

### Evaluation of the cellular uptake of trio-coated PEI and DI-NPs

The hMSCs were seeded onto a microscope coverglass (18 mm diameter; 0111580, Marienfeld) and treated with trio-coated PEI and DI-NPs for 0.5, 1, and 4 h. The uptake of trio-coated PEI and DI-NPs was visualized by fluorescence confocal microscopy (LSM 880 META, Zeiss). The uptake of trio-coated PEI and DI-NPs was also visualized by staining endosomes. The NP-treated hMSCs were exposed to the Cell Light® Reagent BacMam 2.0 (2 μL per 10,000 cells), which stains early endosomes, for 16 h at 37 °C and visualized by fluorescence confocal microscopy.

### Evaluation of chondrogenic and osteogenic markers

The mRNA and protein were extracted from hMSCs cultured in two-dimensional and 3D systems at 1, 3, 7, 14, and 21 days after transfection. Thereafter, mRNA and protein expression were evaluated by RT-PCR and Western blotting, respectively.

### Microarray analysis

Total RNA was extracted using TRIzol (Invitrogen), and its quality was determined using a spectrophotometer (NanoDrop 2000) and an Agilent Bioanalyzer™ 2100 system. Thereafter, cRNA was synthesized from 10 ng RNA with the WT Pico Reagent Kit (Affymetrix) according to the manufacturer’s instructions. The samples were hybridized onto the Human Gene 2.0 ST Array (Affymetrix) for 17 h at 45 °C according to the manufacturer’s instructions. The arrays were scanned using a GeneChip Scanner 3000 7G (Affymetrix). After normalization, the genes related to differentiation and proliferation were selected and data processing was performed using GeneSpring GX13.1.1.

### Histology and immunofluorescence

The hMSCs cultured in 3D systems were fixed with 4% paraformaldehyde for 1 h. For hMSCs cultured in the 3D system, the pellets were embedded in optimal cutting temperature compound (TISSUE-TEK 4583; Sakura Finetek Inc.) and frozen at −80 °C. The frozen samples were sliced into sections (9 μm thick) using a cryotome (HM 525, MICROM) at −25 °C. All samples were stained with hematoxylin (HHS16, Sigma Aldrich) and eosin (110116, Sigma Aldrich). Staining with Alcian blue (A3157, Sigma Aldrich), Safranin O (HT90432, Sigma Aldrich), and Masson’s trichrome (HT15, Sigma Aldrich) was performed to evaluate chondrogenic differentiation. All staining was performed according to the manufacturer’s instructions. Immunofluorescence was performed under humidified conditions using primary antibodies against SOX9, type 1 collagen (COLI), type 2 collagen (COLII), and matrix metallopeptidase (MMP) 3. Thereafter, the samples were stained with fluorescently labeled secondary antibodies (1:500; Thermo Scientific), incubated with 4,6-diamidino-2-phenylindole (DAPI) for 10 min to stain the nuclei, and visualized by fluorescence confocal microscopy (LSM 880 META, Zeiss).

### Statistical analysis

All data are shown from at least three independent experiments performed in triplicate. Statistical comparisons were carried out by the Student’s *t* test and one-way analysis of variance (ANOVA). Probability less than 0.05 was considered statistically significant.

## Results

### Preparation and characterization of trio-coated PEI and DI-NPs

**Fig. 1:**
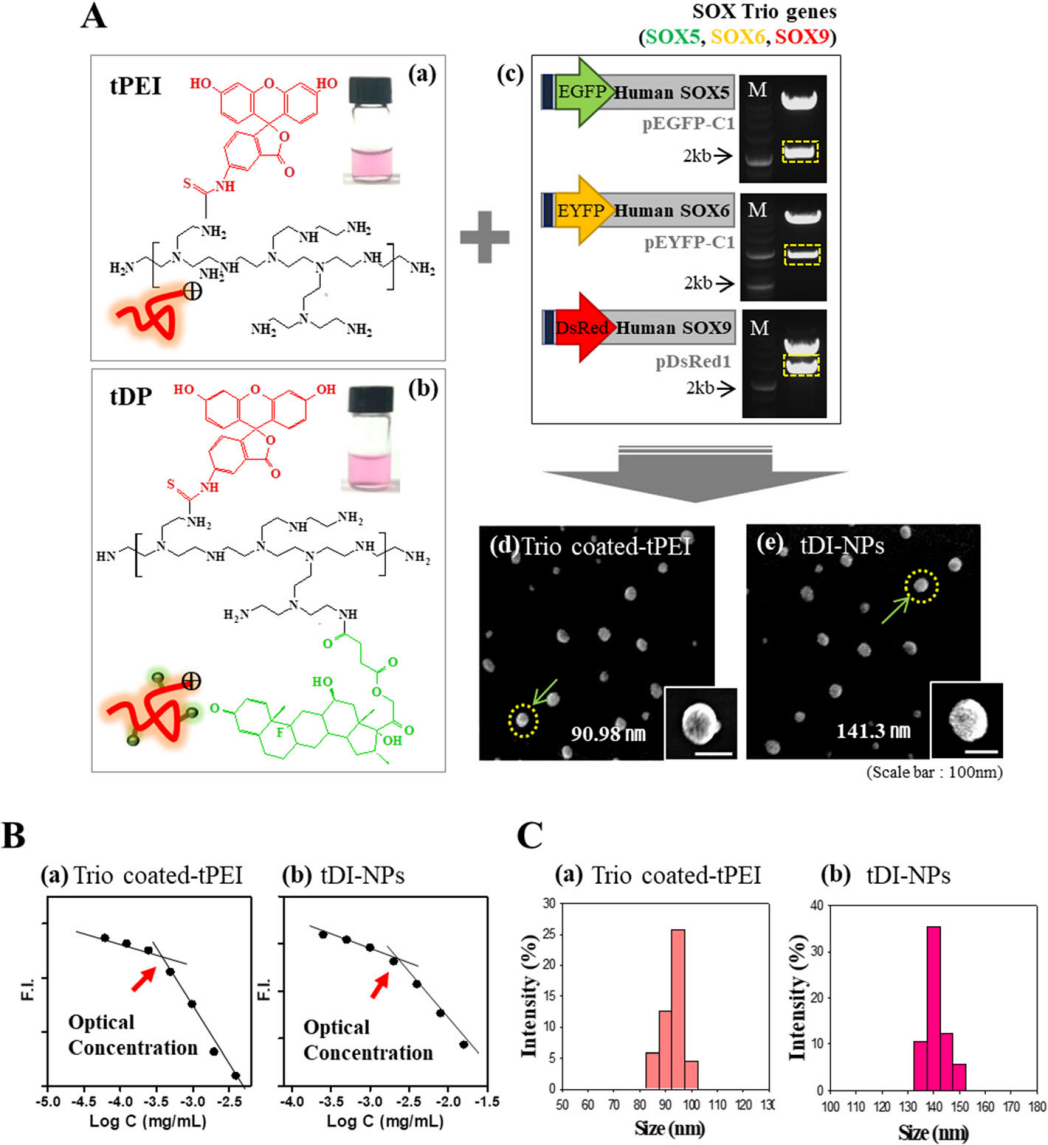
Preparation and characterization of trio-coated PEI and DI-NPs. **a** Structures of polyethyleneimine (PEI; panel a) and dexamethasone-conjugated polyethyleneimine (DEX/PEI; panel b). Representative PCR analysis of SOX5, SOX6, and SOX9 pDNAs (panel c). SEM analysis of trio-coated PEI (panel d) and differentiation-inducing nanoparticles (DI-NPs; panel e). **b** Optimal concentrations of trio-coated PEI (panel a) and DI-NPs (panel b) for micelle formation. **c** Analysis of size of trio-coated PEI (panel a) and DI-NPs (panel b) by dynamic light scattering. EGFP enhanced green fluorescent protein, EYFP enhanced yellow fluorescent protein

Here, bPEI was conjugated with TRITC to generate PEI, which was subsequently conjugated with DEX to generate DEX/PEI. Thereafter, DEX/PEI were complexed with SOX5, SOX6, and SOX9 pDNAs to generate DI-NPs. Figure 1a shows the molecular structures of PEI and DEX/PEI, PCR analysis of pDNAs, and SEM images of SOX5/6/9 (trio)-coated PEI and DI-NPs. DEX and PEI formed micelles at certain concentrations (Fig. 1b). The hydrophobic moiety of DEX was hidden within the core, while the hydrophilic moiety of PEI was exposed on the outer shell. Thus, PEI could be complexed with specific materials. Fluorescent dye-conjugated bPEI makes it possible to trace the nanoparticle location in vitro and in vivo with various tools, including confocal laser microscopy and xenogen. In SEM analysis, trio-coated PEI and DI-NPs had diameters of 90 and 141 nm, respectively (Fig. 1a, panels d and e). The mean diameter of trio-coated PEI and DI-NP was 85.9 ± 10 nm and 138.7 ± 11 nm, respectively. This size difference may be due to DEX conjugation in the latter (Fig. 1c, panels a and b). Complexation with negatively charged pDNA changed the surface charge of DI-NPs (Additional file 1: Figure S1A). To confirm the complexation of PEI and pDNA, a gel retardation assay was performed (Additional file 1: Figure S1B). All pDNA was complexed when more than 1.5 μg of PEI or more than 2.5 μg of DEX/PEI was used. Tight complexation did not occur with 1.5 μg of DEX/PEI, which may be due to DEX conjugation. Thus, more DEX/PEI than PEI was used for complexation with pDNA.

### Evaluating cytotoxicity and uptake efficiency of trio-coated PEI and DI-NPs into hMSCs

**Fig. 2:**
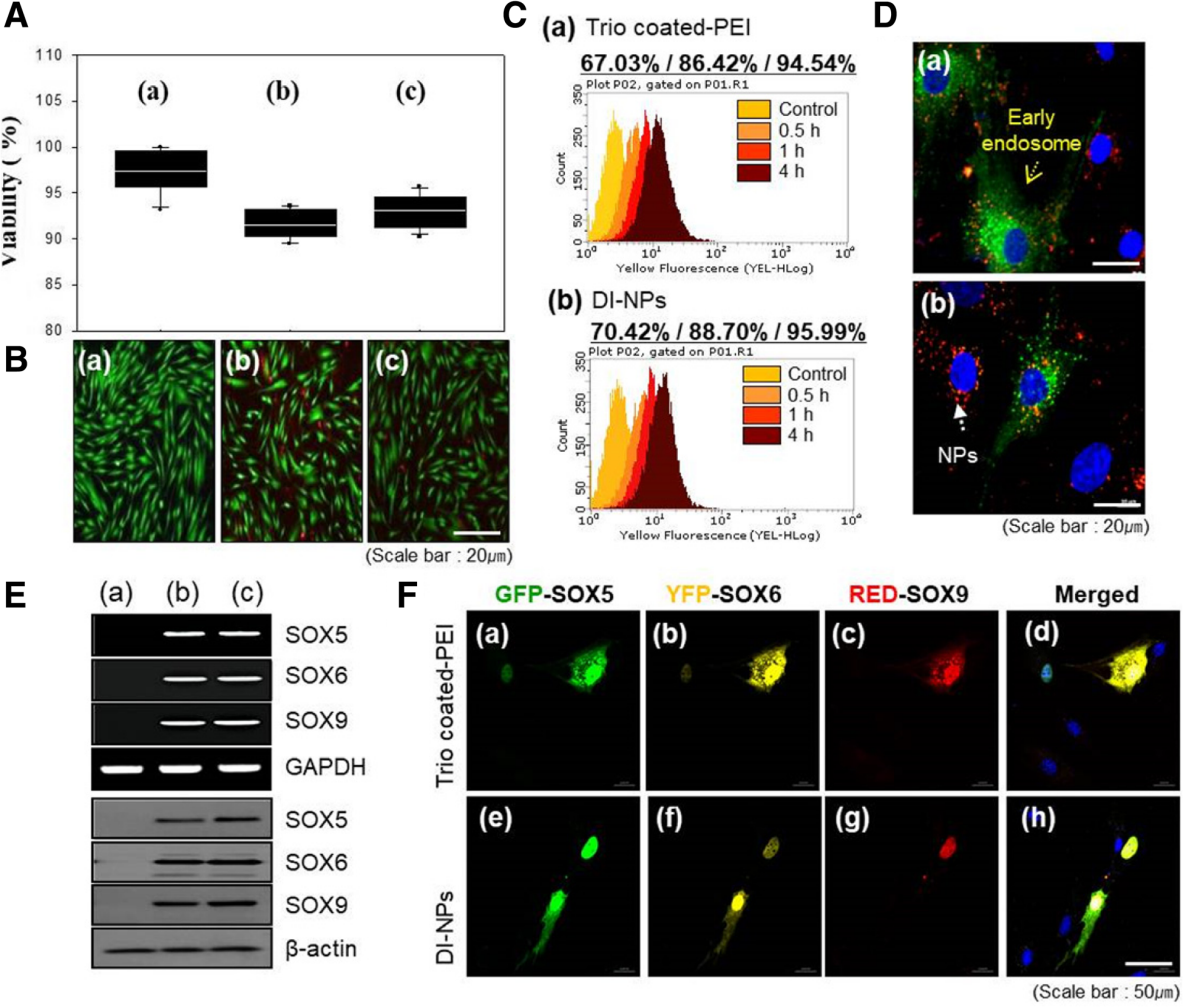
Evaluation of cytotoxicity, uptake efficiency of nanoparticles (NPs), and expression levels of delivered genes. **a**, **b** Assessment of the cytotoxicity of trio-coated polyethyleneimine (PEI) and differentiation-inducing nanoparticles (DI-NPs) by CCK8 and Live/Dead assays in hMSCs transfected with nothing (panel a), trio-coated PEI (panel b), and DI-NPs (panel c). **c**, **d** Assessment of the cellular uptake of trio-coated PEI (panel a) and DI-NPs (panel b) into hMSCs over time by FACS and fluorescence microscopy. **e** RT-PCR and Western blot analysis of SOX5, SOX6, and SOX9 in hMSCs transfected with nothing (column a), trio-coated PEI (column b), and DI-NPs (column c). **f** Confocal microscopy analysis of SOX5, SOX6, and SOX9 in hMSCs transfected with PEI and DI-NPs complexed with SOX5 (panels a and e), SOX6 (panels b and f), SOX9 (panels c and g), and merged images (panels d and h). GFP green fluorescent protein, YFP yellow fluorescent protein

Cytotoxicity was evaluated by CCK8 and live/dead assays (Fig. 2a, b). Following treatment with trio-coated PEI or DI-NP, more than 90% of the hMSCs survived (Fig. 2a). In the live/dead assay, red labeling, indicative of dead hMSCs, was not clearly observed in nontreated or DI-NP-treated cultures (Fig. 2b, panels a and c), but was often observed in trio-coated PEI-treated cultures (Fig. 2b, panel b). This finding indicates that DEX/PEI is a much safer gene delivery carrier than PEI for hMSCs, and the cytotoxicity of DEX/PEI depends on its amount, as shown by FACS analysis (Additional file 1: Figure S2). Side scatter (SSC) and forward scatter (FSC) show the cell state and cell size, respectively. As the amount of DEX/PEI increased, the population of cells with higher SSC values increased in the dot plot, suggesting that dead cells are increased due to the toxicity of high concentrations of DEX/PEI. Using 1 μg of DEX/PEIs, the cytotoxicity of DEX/PEI was evaluated in a time sequence by FACS analysis (Additional file 1: Figure S3). The results showed that DEX/PEI did not affect the cell state until 48 h. FACS analysis demonstrated that trio-coated PEI and DI-NP were internalized into hMSCs with time, and finally up to 95% of hMSCs were transfected (Fig. 2c, panels a and b) by 4 h post-transfection. Thus, these gene carriers readily entered the hMSCs. The hMSCs were imaged by confocal laser microscopy (Fig. 2d). Red labeling, indicative of NPs, and green labeling, indicative of early endosomes, were clearly colocalized (Fig. 2d, panels a and b). Thus, both gene carriers entered hMSCs via endocytosis.

### Evaluating expression efficiency of trio-coated PEI and DI-NPs

The hMSCs transfected with trio-coated PEI or DI-NPs were evaluated by Western blotting and RT-PCR (Fig. 2e). The mRNA and protein expression of SOX5, SOX6, and SOX9 were detected in cells transfected with either gene carrier (Fig. 2e, panels b and c), demonstrating that all three SOX genes were readily delivered into hMSCs, transcribed, and translated. The protein expression of enhanced green fluorescent protein (EGFP)-tagged SOX5, enhanced yellow fluorescent protein (EYFP)-tagged SOX6, and DsRed-tagged SOX9 in hMSCs was evaluated by confocal laser microscopy using specific antibodies. The results demonstrated that SOX5, SOX6, and SOX9 proteins were expressed in hMSCs (Fig. 2f). Merged images showed that all three SOX genes were simultaneously expressed (Fig. 2f, panels d and h). Moreover, the SOX proteins were colocalized, indicating that all three genes were simultaneously delivered into hMSCs. Together, these results demonstrate that fluorescently labeled SOX5, SOX6, and SOX9 were co-delivered into hMSCs using either gene carrier. The uptake of PEI and DI-NPs was examined for a short period (Additional file 1: Figure S4). In present study, the PEI and DI-NPs showed localization in the cytosol of hMSCs in a short time. The hMSCs were transfected with pDNA harboring green fluorescent protein (GFP) using various amounts of PEI or DEX/PEI (Additional file 1: Figure S5). Following transfection with 1, 3, or 5 μg PEI, 20% of the hMSCs were GFP-positive (Additional file 1: Figure S5A, panels a–c). However, 39%, 25%, and 34% of hMSCs were GFP-positive following transfection with 1, 3, and 5 μg DEX/PEI (Additional file 1: Figure S5A, panels d–f). DEX/PEI has the higher transfection efficiency than PEI due to its compacted morphology and ease of endocytosis into cells, resulting from its hydrophobic moiety from dexamethasone. Therefore, 1 μg DEX/PEI was sufficient to deliver GFP pDNA into hMSCs. GFP protein expression was detected by Western blotting (Additional file 1: Figure S5B). GFP expression differed according to the type and amount of gene carrier. The GFP band was denser for hMSCs transfected using DEX/PEI than for those transfected using PEI. GFP protein expression decreased as the amount of DEX/PEI increased. The quantification of GFP expression in hMSCs confirmed that GFP expression decreased as the amounts of PEI and DEX/PEI increased (Additional file 1: Figure S5B). The fluorescence microscopy analysis of GFP expression in hMSCs is presented in Additional file 1 (Figure S6). Consistent with the FACS and Western blot assays, the fluorescence intensity of GFP was higher in hMSCs transfected using DEXPEI than in those transfected using PEI (Additional file 1: Figure S6, panels d and e).

### Evaluating the inductive level of chondrogenic differentiation depending on time over 21 days

**Fig. 3:**
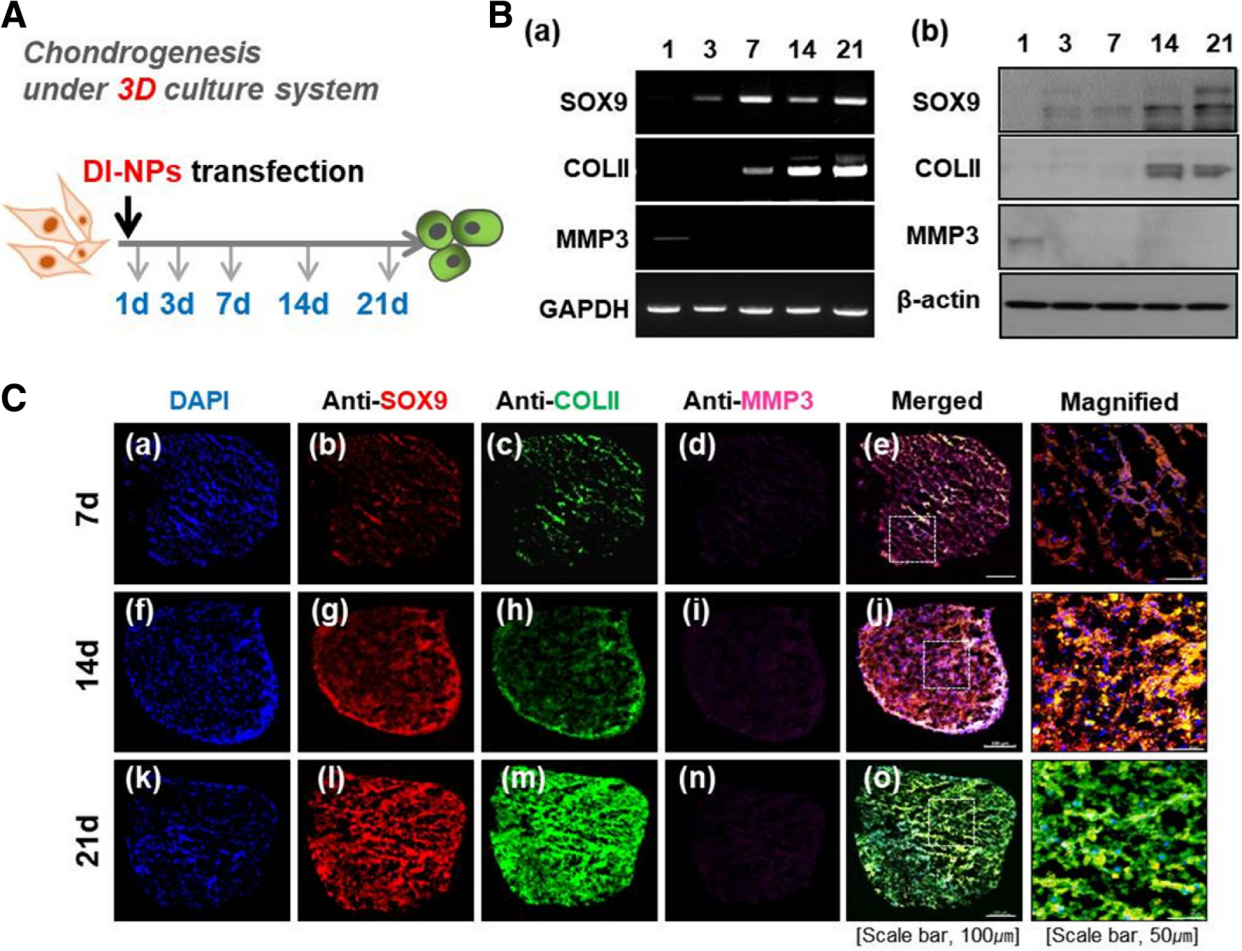
Evaluation of the expression level of chondrogenesis-related markers at various time points after the transfection of hMSCs. **a** Brief schematic of the 3D culture of hMSCs following the transfection of differentiation-inducing nanoparticles (DI-NPs). **b** RT-PCR (panel a) and Western blot analysis (panel b) of chondrogenesis-related markers in hMSCs transfected with DI-NPs. **c** Immunohistological analysis of hMSCs at 7 days (panels a–e), 14 days (panels f–j), and 21 days (panels k–o) after the transfection of DI-NPs. *n* = 5. COL collagen, MMP matrix metallopeptidase

The assay used to assess chondrogenic differentiation of hMSCs following transfection of the three SOX genes is illustrated in Fig. 3a. At 1, 3, 7, 14, and 21 days after transfection, the mRNA and protein expression of SOX9 and COLII, which are differentiation-related markers, and MMP3 was evaluated by RT-PCR and Western blotting (Fig. 3b). MMP3 was barely expressed at 1 day and was not detected at subsequent time points. COLII expression was high from 7 days after transfection (Fig. 3b). Upon transfection of the three SOX genes, the expression of SOX9 and COLII greatly increased. Moreover, the decrease in MMP3 expression and the increases in SOX9 and COLII expression upon transfection of hMSCs were time-dependent. MMP3, SOX9, and COLII expression was assessed by immunofluorescence microscopy at various time points after the transfection of hMSCs with DI-NPs (Fig. 3c). Pink labeling, indicative of MMP3, was not clearly observed in hMSCs (Fig. 3c, panels d, i, and n). Red labeling, indicative of SOX9, was not observed at 7 days (Fig. 3Cb), but was clearly observed at 21 days (Fig. 3c, panel l). Green labeling, indicative of COLII, was bright at 21 days (Fig. 3c, panel k). Thus, transfection of DI-NPs induced differentiation of hMSCs into mature chondrocytes.

### Evaluating differentiation- and proliferation-related markers in hMSCs transfected with DI-NPs by microarray analysis

**Fig. 4:**
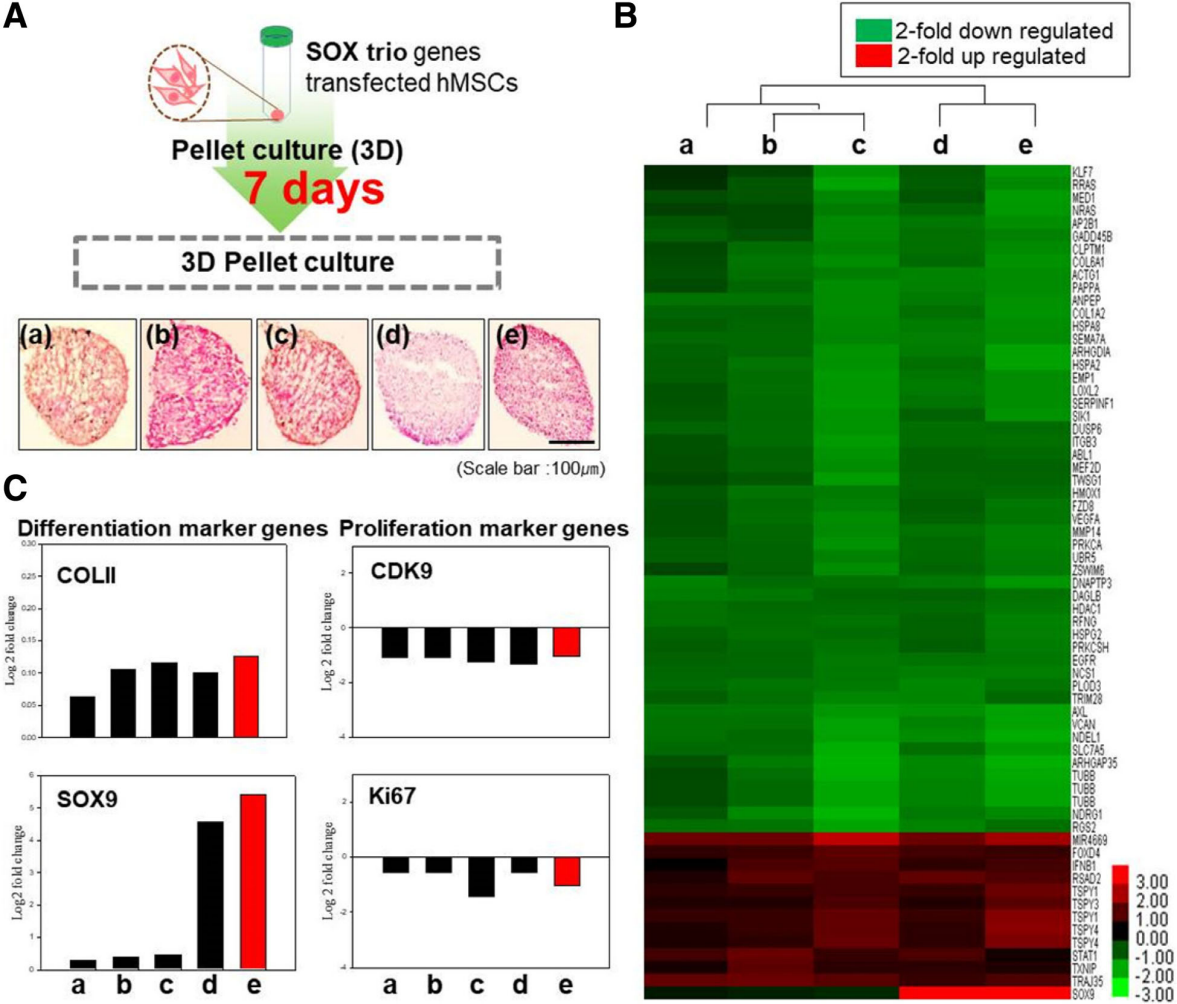
Microarray analysis of hMSCs transfected with DI-NPs and cultured for 7 days in a 3D system. **a** Schematic illustration of the microarray analysis. **b** Heatmap showing the expression of genes related to differentiation and proliferation in each group of transfected human mesenchymal stem cells (hMSCs) relative to nontreated hMSCs. **c** Log2 fold-changes in the gene expression of COLII, SOX9, PCNA, and Ki67 in each group of transfected hMSCs relative to nontreated hMSCs: hMSCs transfected with DEX/PEI only (panel a), SOX5-complexed DEX/PEI (panel b), SOX6-complexed DEX/PEI (panel c), SOX9-complexed DEX/PEI (panel d), and SOX5/6/9 (trio)-complexed DEX/PEI (DI-NPs) (panel e). *n* = 5. COL collagen

The hMSCs were cultured in a 3D system for 7 days after transfection (Fig. 4a), and then microarray analysis was performed (Fig. 4b). The robust multi-array average method was employed, and the data are presented as a scatter plot. The mRNA expression of SOX9 was upregulated more than twofold in hMSCs transfected with the three SOX genes. Quantification demonstrated that the mRNA levels of the differentiation-related markers SOX9 and COLII were increased in hMSCs transfected with the three SOX genes (Fig. 4c, panels d and e), whereas those of the proliferation-related markers CDK9 and Ki67 were not. Thus, hMSCs transfected with the three SOX genes stopped proliferating and underwent chondrogenic differentiation.

### Evaluating mRNA expression level of differentiation-related genes in hMSCs transfected with DI-NPs by microarray analysis

**Fig. 5:**
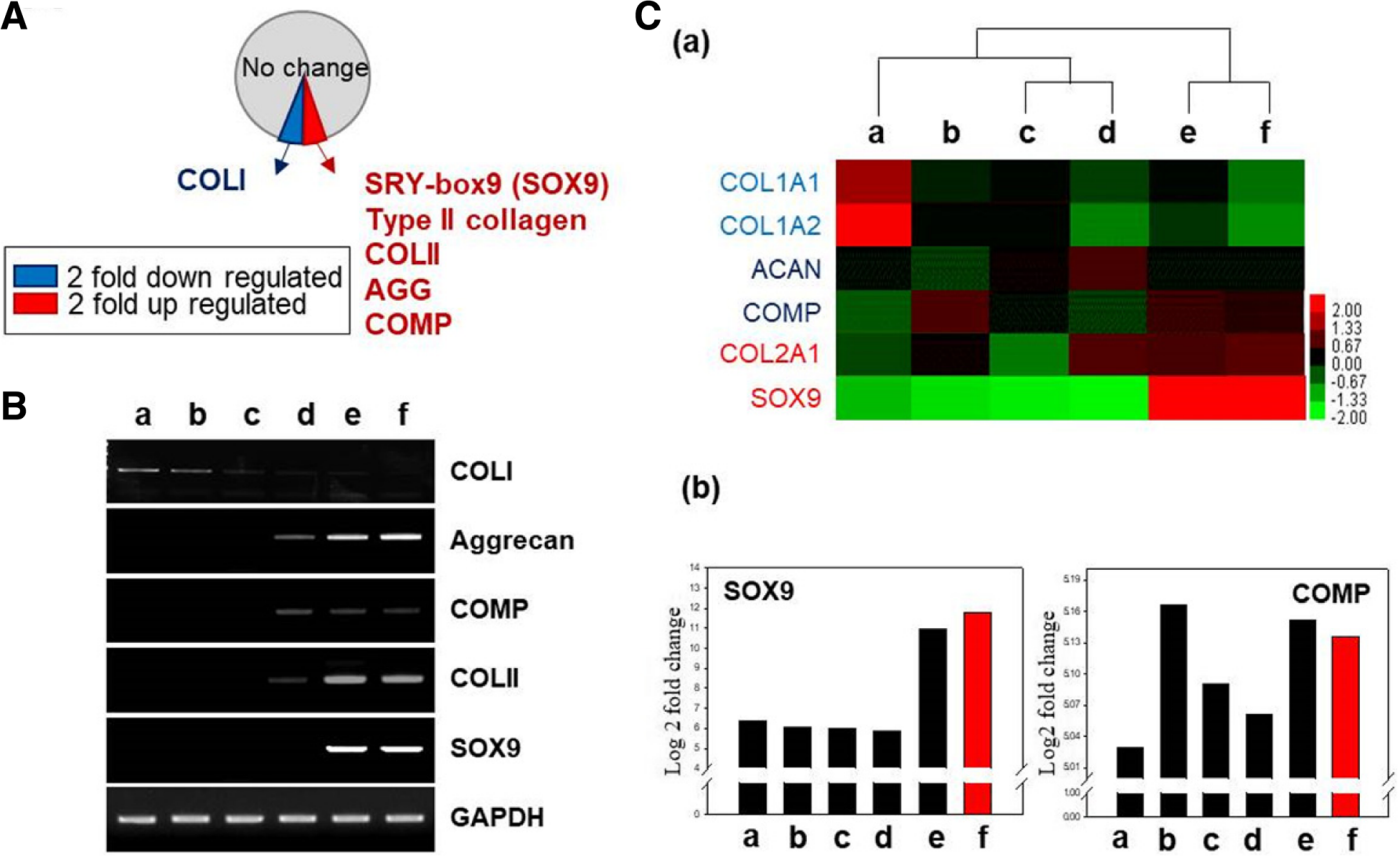
The mRNA expression level of differentiation-related genes in transfected hMSCs. **a** Pie chart of the microarray data show that SOX9 and COLII were upregulated and MMP3 and COLI were downregulated in hMSCs. **b** RT-PCR analysis of chondrogenesis-related markers. The hMSCs transfected with nothing (panel a), DEX/PEI only (panel b), SOX5- complexed DEX/PEI (panel c), SOX6-complexed DEX/PEI (panel d), SOX9-complexed DEX/PEI (panel e), and SOX5/6/9 (trio) conjugated DEX/PEI (DI-NPs) (panel f). **c** Heatmap showing the expression of COL1A1, COL1A2, Aggrecan (ACAN), COMP, and SOX9 in each group of hMSCs relative to nontreated hMSCs (panel a). Log2 fold-changes in the expression of SOX9 and COMP in each group of hMSCs relative to that in nontreated hMSCs (panel b). *n* = 5. COL collagen

The expression of genes related to osteogenesis and chondrogenesis was evaluated in hMSCs transfected with one or all three SOX genes (Fig. 5). A pie chart of the microarray data is presented in Fig. 5a. Chondrogenesis-related marker genes such as SOX9, COLII, Aggrecan, and cartilage oligomeric matrix protein (COMP) were upregulated, whereas the osteogenesis-related gene COLI was downregulated, in hMSCs transfected with DI-NPs (Fig. 5a). RT-PCR analysis demonstrated that Aggrecan, COMP, COLII, and SOX9 were expressed in hMSCs transfected with SOX9 alone or with all three SOX genes (Fig. 5b, panels e and f). However, COLI was not expressed in either group of hMSCs. Thus, the transfection of SOX9 or all three SOX genes induced chondrogenesis and inhibited the osteogenesis of hMSCs. Microarray analysis of the various groups of hMSCs was performed (Fig. 5c, panel a). SOX9, a marker of the early stage of chondrogenesis, was highly expressed, whereas COMP and COLII, markers of the final stage, were not. This difference is because the cells were only cultured for 1 week after transfection. We quantified the mRNA expression of SOX9 and COMP (Fig. 5c, panel b). SOX9 was highly expressed in hMSCs transfected with SOX9 alone or all three SOX genes. However, the expression of COMP did not significantly differ between the groups of hMSCs. Thus, the culture of hMSC masses for 1 week following transfection was insufficient to complete chondrogenesis.

**Fig. 6:**
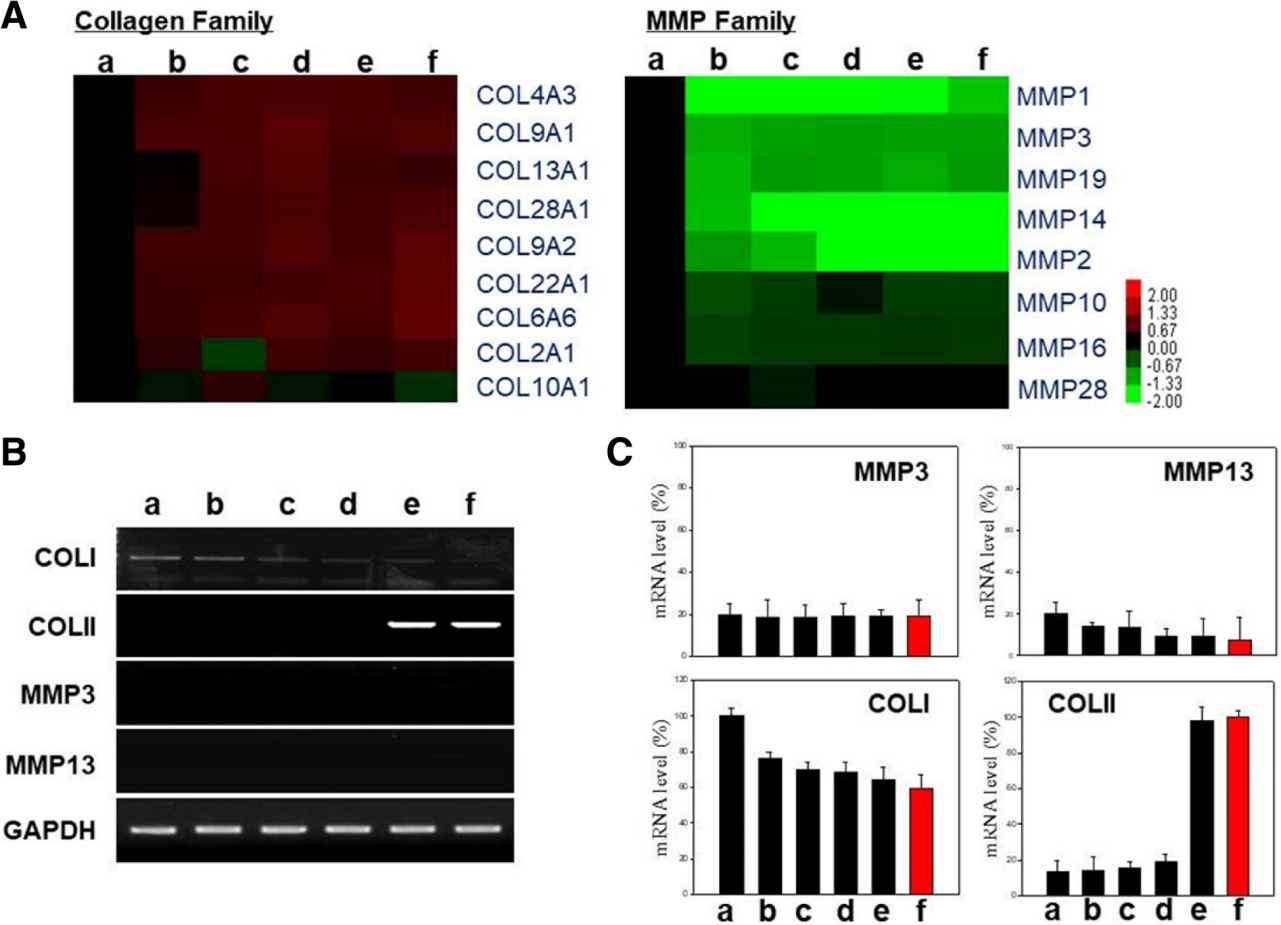
Expression level of mRNA of members of the collagen and MMP families in transfected hMSCs. **a** Heatmap showing the expression of various members of the collagen and MMP families in each group of hMSCs relative to that in nontreated hMSCs. **b** RT-PCR analysis of COLI, COLII, MMP3, and MMP13 in each group of hMSCs. **c** Bar graphs of the mRNA levels of COLI, COLII, MMP3, and MMP13. The hMSCs transfected with nothing (panel a), DEX/PEI only (panel b), SOX5-complexed DEX/PEI (panel c), SOX6-complexed DEX/PEI (panel d), SOX9-complexed DEX/PEI (panel e), and SOX5/6/9 (trio) complexed DEX/PEI (DI-NPs) (panel f). *n* = 5. COL collagen, MMP matrix metallopeptidase

Microarray analysis was performed for genes belonging to the collagen and MMP families (Fig. 6a). The expression of chondrogenesis-related collagen isoforms was increased in hMSCs transfected with SOX9 alone or all three SOX genes. Thus, both transfections triggered the gene expression of specific collagen isoforms, which induced hMSCs to differentiate. MMPs play a major role in cell proliferation, migration, differentiation, angiogenesis, apoptosis, and host defense. However, MMP genes were not expressed in hMSCs transfected with SOX9 alone or all three SOX genes. Chondrogenesis-related markers were analyzed by RT-PCR (Fig. 6b). MMP3 and MMP13, which are not related to differentiation, and COLI, an osteogenesis-related marker, were not expressed in hMSCs transfected with SOX9 alone or all three SOX genes (Fig. 6b, panels e and f). However, the chondrogenesis-specific marker COLII was highly expressed in both groups of hMSCs. The quantification of mRNA expression confirmed that MMP3 and MMP13 levels did not differ among the various groups of hMSCs (Fig. 6c). However, COLI and COLII expression was almost twofold lower and approximately sevenfold higher, respectively, in hMSCs transfected with SOX9 alone and all three SOX genes than in control hMSCs and those transfected with SOX5 or SOX6 alone.

### Evaluating the inductive level of chondrogenic differentiation in a 3D system

**Fig. 7:**
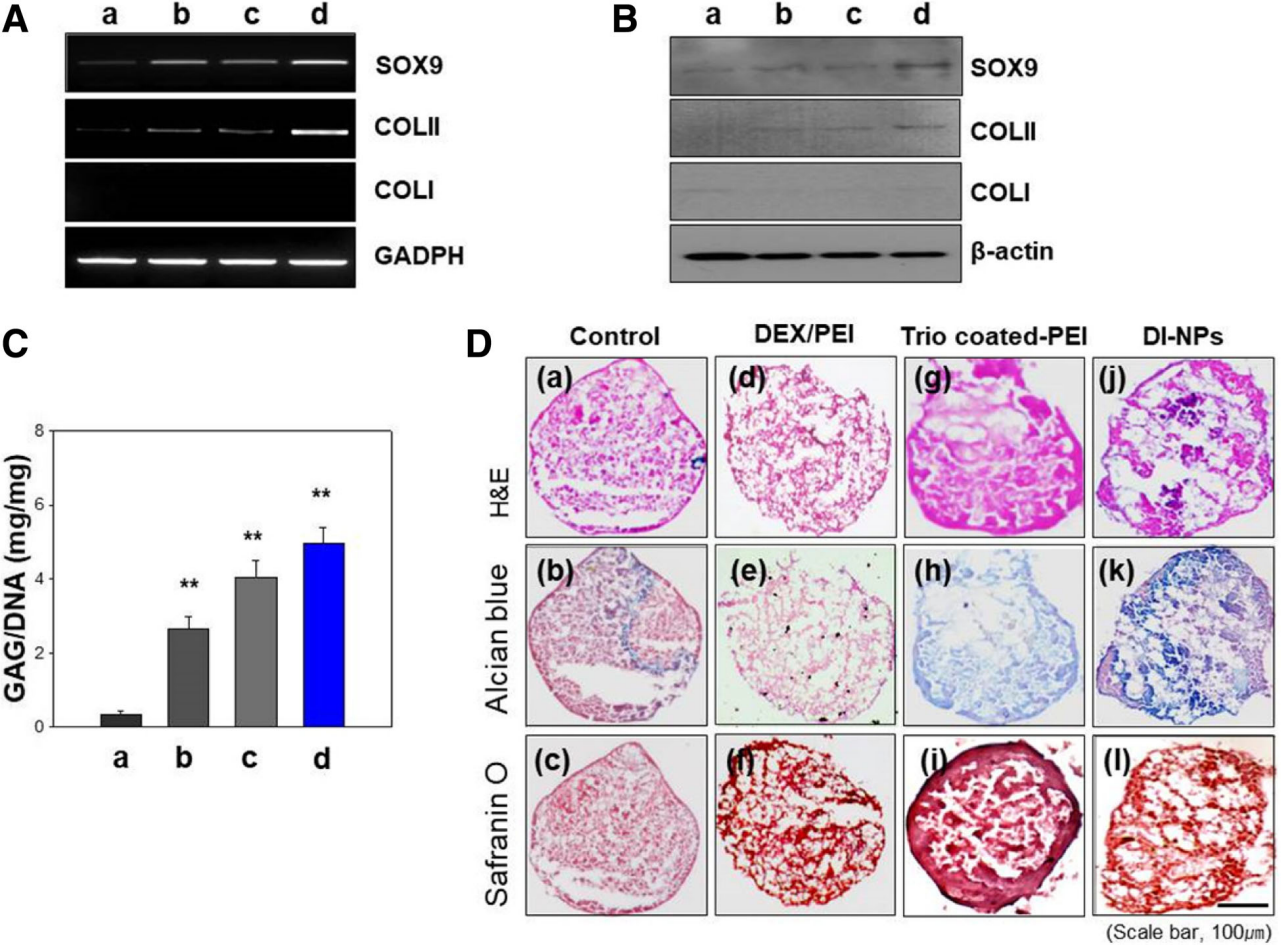
Assessment of induction levels of chondrogenic differentiation of hMSCs cultured in a 3D system. **a**, **b** RT-PCR and Western blot analysis of SOX9, COLII, and COLI. **c** Glycosaminoglycan (GAG) assay hMSCs transfected with nothing (panel a), DEX/PEI only (panel b), trio-coated PEI (panel c), and DI-NPs (panel d). **d** Histological analysis of non-treated (panels a–c), Dexamethasone-conjugated polyethyleneimine (DEX/PEI)-treated (panels d–f), trio-coated PEI treated (panels g–i), and differentiation-inducing nanoparticle (DI-NP)-treated (panels j–l) hMSCs cultured in a 3D system. *n* = 5. **p < 0.01. COL collagen, H&E hematoxylin and eosin

The hMSCs were transfected with all three SOX genes using various carriers and subsequently cultured in a 3D system for 3 weeks. The transfection efficiency was determined by RT-PCR and Western blotting (Fig. 7a, b). The expression of the chondrogenesis-related marker genes SOX9 and COLII was higher in hMSCs transfected with DI-NPs than in those transfected with trio-coated PEI or DEX/PEI (Fig. 7a, panel d). The expression of the osteogenic marker COLI was not detected in hMSCs transfected with DI-NPs. Furthermore, the expression of the hypertrophy marker, collagen type X (COLX), in hMSCs transfected with DI-NPs was lower than hMSCs transfected with nothing (Additional file 1: Figure S7). Chondrogenesis-related markers were also expressed at the protein level (Fig. 7b). SOX9 and COLII proteins were expressed in hMSCs transfected with DI-NPs. Although these proteins were also expressed in hMSCs transfected with trio-coated PEI or DEX/PEI, their expression levels were not as high as in hMSCs transfected with DI-NPs. The glycosaminoglycan (GAG) content, an indicator of chondrogenesis, was normalized to the DNA content. The normalized GAG content of hMSCs transfected with DI-NPs was higher than that of control hMSCs and those transfected with trio-coated PEI or DEX/PEI (Fig. 7c). The hMSCs transfected with DI-NPs were brightly stained with Alcian blue and Safranin O, which label proteoglycans, whereas control hMSCs and those transfected with other gene carriers were not (Fig. 7d). Immunohistological analysis was performed for COLII, SOX9 (both endogenous and exogenous), and COLI in transfected hMSCs (Additional file 1: Figure S8). The masses of hMSCs transfected with DI-NPs were compact, had a highly distributed morphology, and were brightly stained green and red, representing COLII and SOX9, respectively. In contrast, the masses of control hMSCs and those transfected with DEX/PEI or trio-coated PEI were not brightly stained green or red. Thus, the transfection of DI-NPs induced chondrogenesis of hMSCs.

## Discussion

The efficient delivery of chondrogenesis-related genes to stem cells is one of the important aims for the treatment of cartilage defects in stem cell therapy [27]. There are few studies on cell signaling involved in the differentiation of mesenchymal stem cells into chondrocytes, so it is important to study the mechanism of chondrogenic differentiation. Nanoparticles have already been fabricated and studied a lot for this aim, considering biocompatibility, biodegradability, and efficiency of uptake into cells. In this regard, DI-NPs were designed and fabricated for inducing chondrogenic differentiation. DI-NPs are composed of DEX/PEI and pDNA of the SOX trio (SOX5, SOX6, and SOX9).

Although DEX/PEI contains a lower amount of PEI in the same volume compared with PEI, DEX/PEI has a higher transfection efficiency. DEX/PEI improved transfection efficiency compared with PEI alone due to two favorable effects. First, DEX when conjugated to PEI led to nuclear entry of DEX/PEI being more effective in cells. Second, in an aqueous solution, hydrophobic DEX would be recruited in a core and make hydrophilic PEI exist on the surface of the structure. Therefore, the higher density of the positive charge is represented on the surface of DEX/PEI, resulting in better complexation with pDNA. This means that the compressed and stable structure of DEX/PEI is effective in delivering pDNA to MSCs.

In addition, SOX9, constituting a DI-NP, is an essential transcription factor while, though not necessarily required in chondrogenesis, SOX5 and SOX6 cooperate with SOX9 to activate the expression of COLII and Aggrecan [28]. This strongly suggests that the SOX trio proteins fulfill most of their functions in differentiated chondrocytes cooperatively rather than independently of one another.

Here, overexpressed SOX9, along with SOX5 and SOX6 by DI-NPs, increased the expression of COLII, Aggrecan, and COMP. An interaction between these extracellular matrix (ECM) proteins in differentiated chondrocytes provided a proper environment for the progression of chondrogenesis. Moreover, the genetic level of stem cells induced by DI-NPs or those noninduced were screened with genes related to chondrogenic differentiation. Chondrogenic differentiation-related genes (SOX9, COLII, Aggrecan, and COMP) were upregulated and osteogenic differentiation-related genes (COLI) were downregulated. Furthermore, the increase in collagen family genes and the decrease in MMP family genes observed in hMSCs induced chondrogenic differentiation. MMPs are proteolytic enzymes for the degradation of ECM proteins [29]. Thus, reduced MMP3 expression and increased expression of ECM-related genes increased the levels of proteoglycans, suggesting some degree of differentiation into chondrocytes, indicating that DI-NPs induced chondrogenic differentiation of hMSCs.

This study increases the knowledge on SOX5, SOX6, and SOX9, uncovering molecular networks for the regulation of chondrogenesis. It has also raised new questions for further investigation. For instance, we need to set a framework for complementary studies to establish the actions of the SOX trio at the stages of chondrocyte differentiation. We also need to uncover transcription factors that are likely to functionally interact positively or negatively with the SOX trio in chondrocytes. In addition, these data represent a comprehensive examination of gene expression across the process of chondrogenic differentiation and we attempted to identify specific genes for differentiation using microarray analysis. The gene-based investigations presented here, as well as using additional approaches to represent induction of differentiation, will be helpful to understand the processes of differentiation. Furthermore, through detailed studies of microarray analysis and based on a comprehensive understanding of the factors related to the cartilage tissue engineering process, further research is needed on systemic induction of chondrogenic differentiation by controlling the cell proliferation (cell cycle), inhibition of osteogenesis, and promoting induction of chondrogenesis.

## Conclusions

The present study demonstrates that DI-NPs can be used to deliver genes into hMSCs. This carrier can be used to develop a nonviral gene delivery system with increased cellular uptake to enhance expression at the mRNA and protein levels. Furthermore, we show that DI-NPs are good tracers for gene delivery and could potentially be used for imaging and tracing using various tools in vitro and in vivo. Additionally, microarray analysis revealed that the DI-NP-transfected hMSCs showed superior expression of genes related to the differentiation and proliferation of cells. Finally, the hMSCs transfected with the trio of SOX genes complexed with DI-NPs were successfully differentiated for chondrogenesis. Thus, the DI-NPs synthesized in the present study will contribute to cartilage tissue engineering.

## Additional file

Additional file 1: Supporting information. (DOCX 557 kb)

## Abbreviations

3D: Three-dimensional
bPEI: Branched polyethyleneimine
CAC: Critical aggregation concentration
COLI: Collagen type 1
COLII: Collagen type 2
COMP: Cartilage oligomeric matrix protein
DEX: Dexamethasone
DEX/PEI: Dexamethasone-conjugated polyethyleneimine
DI-NP: Differentiation-inducing nanoparticle
DMSO: Dimethyl sulfoxide
ECM: Extracellular matrix
FACS: Fluorescence-activated cell sorting
GAG: Glycosaminoglycan
hMSC: Human mesenchymal stem cell
MMP: Matrix metallopeptidase
MSC: Mesenchymal stem cell
MWCO: Molecular weight cut-off
NP: Nanoparticle
pDNA: Plasmid DNA
PEI: Polyethyleneimine
RT-PCR: Reverse-transcription polymerase chain reaction
SEM: Scanning electron microscopy
SOX trio: SOX5, SOX6, and SOX9
TRITC: Tetramethylrhodamine
