# Supplementary material for: Gene expression profiling of chondrogenic differentiation by dexamethasone-conjugated polyethyleneimine with SOX trio genes in stem cells
Source: Stem Cell Res Ther. 2018 Dec 7;9:341. doi: 10.1186/s13287-018-0998-7 (PMC6286596; doi:10.1186/s13287-018-0998-7)
Supplement: Supplementary file 1 — Supporting information. (DOCX 557 kb) [file 13287_2018_998_MOESM1_ESM.docx]

Additional file 1

Gene expression profiling of chondrogenic differentiation by dexamethasone conjugated polyethyleneimine with SOX trio genes in stem cells

Se Won Yi ^a,1^, Hye Jin Kim ^a^, Hyun Jyung Oh ^a^, Heejun Shin ^b^, Jung Sun Lee ^a^, Ji Sun Park ^a, *^ , Keun-Hong Park ^a, *^

^a^Department of Nano-regenerative Medical Engineering, College of Life Science, CHA University, 335, Pangyo-ro, Bundang-gu, Seongnam-si, 134-88, Republic of Korea

^b^ Department of Biotechnology, Catholic University 43-1, Yeokgok 2-dong, Wonmi-gu, Bucheon-si, Gyeonggi-do, 420-743, Republic of Korea

***Co-corresponding Author:** Prof. Keun-Hong Park (E-mail: pkh0410@cha.ac.kr, Phone: +82-31-881-7138), and Prof. Ji Sun Park (E-mail: pjs09@cha.ac.kr, Phone: +82-31-881-7246)

Materials and methods

**1. Materials**

Branched PEI (bPEI, 25 kDa), tetramethylrhodamine (TRITC), dicyclohexylcarbodiimide (DCC), N-hydroxysuccinimide (NHS)**,** Hoechst 33342, and DIO (3,3’-Dioctadecyloxacarbocyanine Perchlorate) were purchased from Sigma Aldrich (St. Louis, MO, USA). DEX hemisuccinate was purchased from Steraloids (Newport, RI, USA). These chemicals were used without further purification. Dialysis membrane (molecular weight cut-off (MWCO), 1,000 Da) was purchased from Spectrum Laboratories Inc. (Rancho Dominguez, CA, USA). Anti-SOX9 antibody (AB5535), type 1 collagen (COLI, MAB3391) and type 2 collagen (COLII, MAB1330) were purchased for Chemicon

**2. Methods**

2-1. Measurement of the charge of NPs

The surface charge of NPs was measured with a Zetasizer Nano ZS apparatus (Malvern, USA). Twenty measurements were conducted per sample.

2-2. Gel retardation assay

Positively charged PEI and DP were neutralized upon complexation with negatively charged pDNA, which retarded DNA mobility on an agarose gel. Non-complexed pDNA was used as a control. The size at which pDNA migrated depended on the amount of PEI used for complexation (0.5–5 µg).

2-3. Cytotoxicity of DI-NPs to hMSCs depending on the amount of DI-NPs and time

A total of 2×10^5^ hMSCs were seeded and 1, 3, 5, 7, 10 µg of DI-NPs were treated for 4 hours. And 1 µg of DI-NPs were treated and confirmed state of cells by FACS. Side Scatter (SSC) and Forward Scatter (FSC) shows cell state and cell size respectively. The higher FSC value is, the more number of dead cells exists.

2-4. Evaluation of cellular uptake of SOX5/6/9 (Trio)-coated PEI and DI-NPs

A total of 2×10^5^ hMSCs were seeded onto a microscope coverglass (18 mmØ, 0111580, Marienfeld) and treated with SOX5/6/9-complexed PEI and DI-NPs for 0.5, 1 and 4 hours. Uptake of Trio coated-PEI and DI-NPs was visualized by fluorescence confocal microscopy (LSM 880 META, Zeiss).

2-5. Immunocytochemistry of the pellet

hMSCs were transfected with optimal concentrations of Trio coated-PEI and DP (so-called DI-NPs) and cultured at 3D culture system for 21 days in serum-free medium. And Immunofluorescence was performed in humidified conditions using primary antibodies against SOX9, type 1 collagen (COLI), and type 2 collagen (COLII). Thereafter, samples were stained with fluorescently labeled secondary antibodies (1:500; Thermo Scientific), incubated with 4, 6-diamidino-2-phenylindole for 10 minutes to stain nuclei, and visualized by fluorescence confocal microscopy (LSM 880 META, Zeiss).

2-6. Real-time PCR for evaluating expression of COL X

A 1 μl of each cDNA was amplified with a 20 μl of mixture containing 1.5 mM MgCl_2_, 20 pM of each primer, and 1× Takara PCR Master Mix (Takara, Otsu, Japan). Samples were subjected to the following conditions in an ExiCycler (Bioneer, Daejeon, Korea): initial denaturation at 94 °C for 10 min followed by 45 cycles of 94 °C for 40 s, 58 °C for 30 s, and 72 °C for 30 s. Relative quantification was calculated via the 2-delta Ct (cycle threshold) method. In order to confirm the amplification of specific transcripts, melting curves were generated at the end of each PCR by cooling the sample to 40 °C and heating them slowly to 95 °C while continuously measuring the fluorescence.


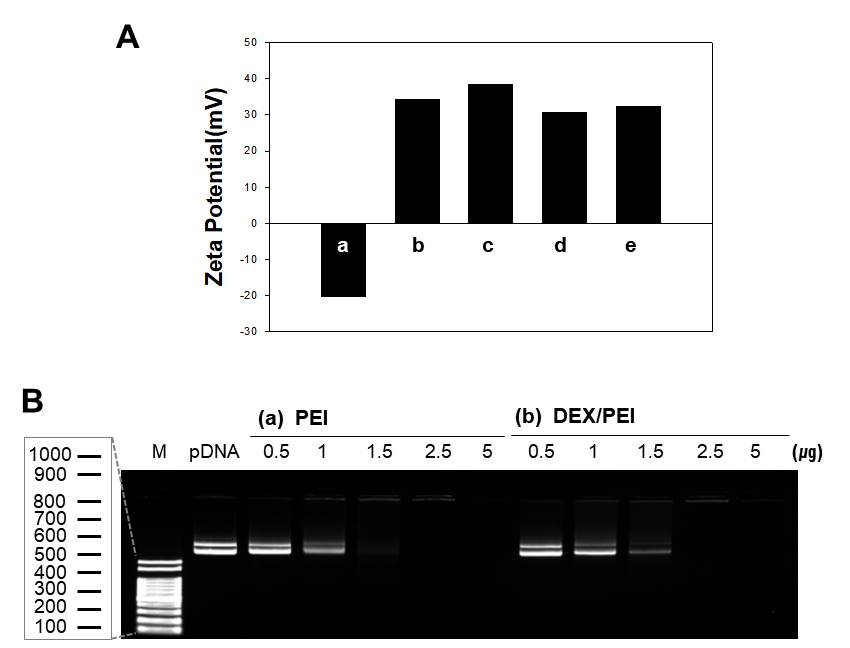


**Figure S1.** ζ-potential of PEI and DEX/PEI and gel retardation assay

Surface charge of pDNA (a), PEI (b), DEX/PEI (c), Trio coated-PEI (d) and DI-NPs (e) was

measured by Dynamic light scattering (DLS) (A). And from 1.5μg of PEI (a) and 2.5 μg of

DEX/PEI (b), NPs were well-complexed with pDNA (B).


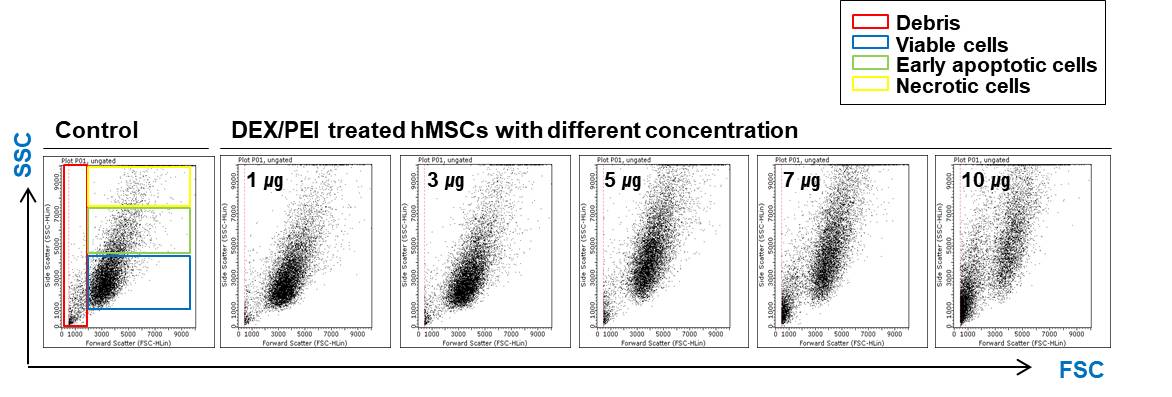


**Figure S2.** Evaluating cytotoxicity of DEX/PEI with different amount to hMSCs by FACS

analysis

It shows that the more DEX/PEI were treated, the higher cytotoxicity was shown


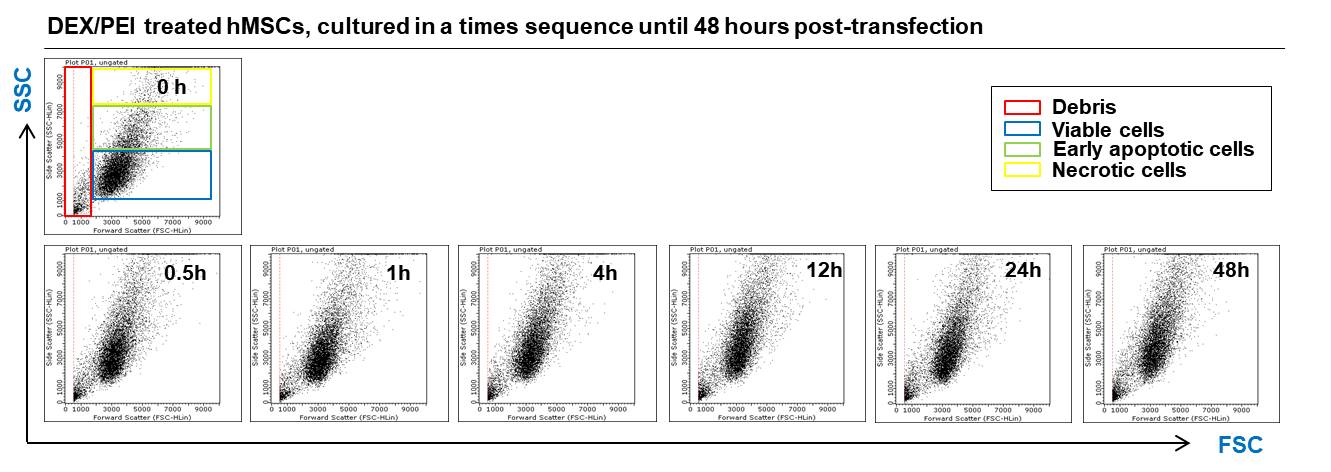


**Figure S3.** Evaluating cytotoxicity of 1 μg of DEX/PEI to hMSCs in a time sequence (From 0

hour to 48 hours) by FACS analysis


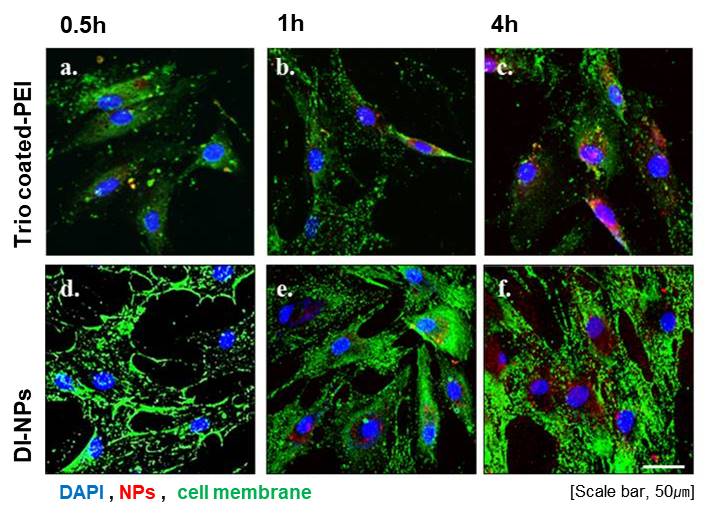


**Figure S4.** Monitoring uptake efficiency of Trio coated-PEI (a-c) and DI-NPs (d-f) into hMSCs

using lipophilic tracer, DIO (3,3’-Dioctadecyloxacarbocyanine Perchlorate) depending on time


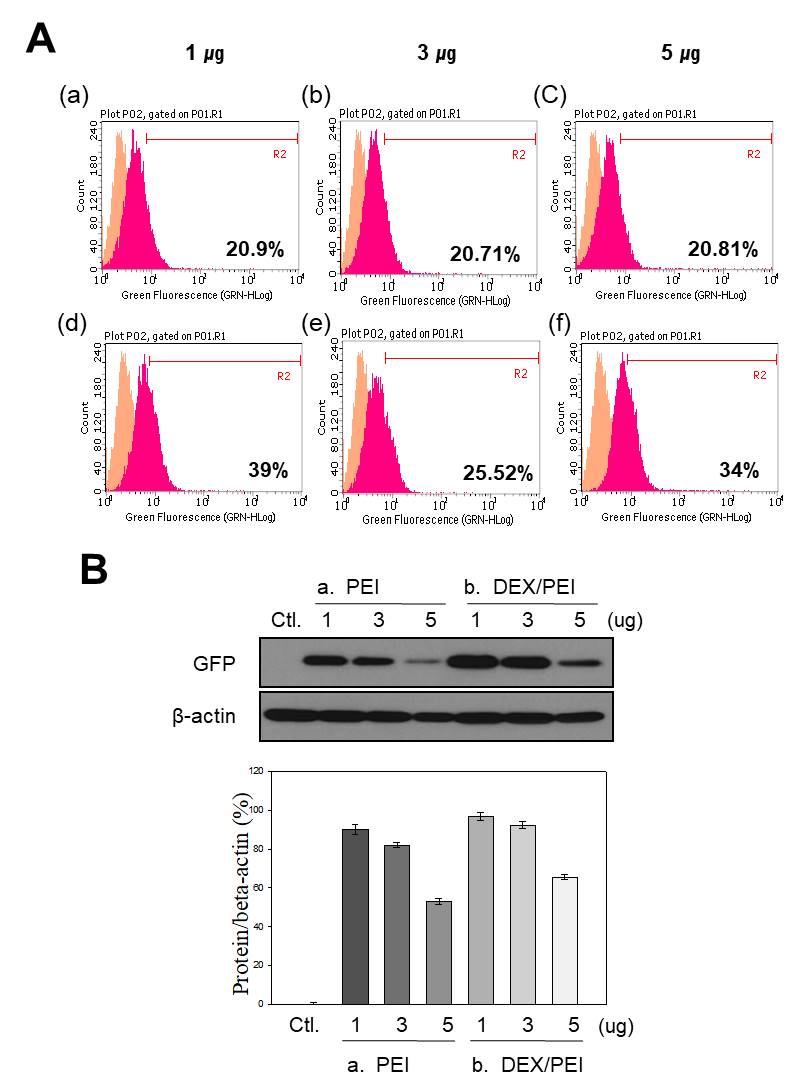


**Figure S5.** Finding proper amount of PEI and DEX/PEI by FACS (A), Western blot analysis (B)

1, 3 and 5 μg of PEI (a-c) and DEX/PEI (d-f) with Green Fluorescent Protein (GFP) pDNA were

transfected to hMSCs. GFP expression level shows DEX/PEI has better delivery efficiency than

PEI, and 1 μg of DEX/PEI is suitable amount for gene delivery.


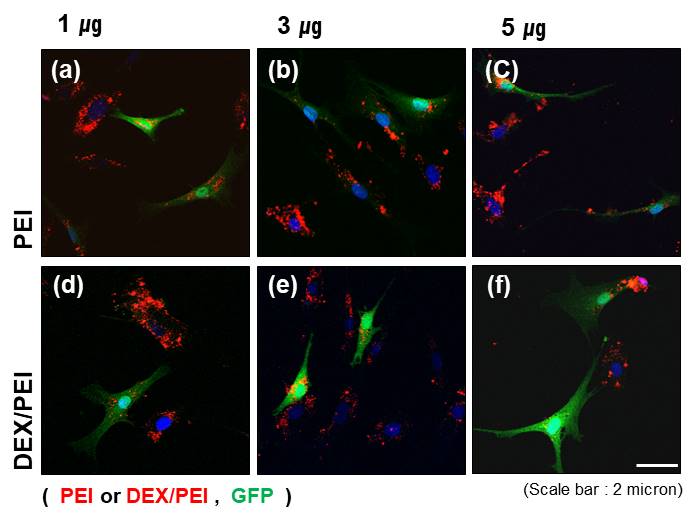


**Figure S6.** Finding proper amount of PEI (a-c) and DEX/PEI (d-f) depending on amount of PEI

by Confocal laser microscope

**
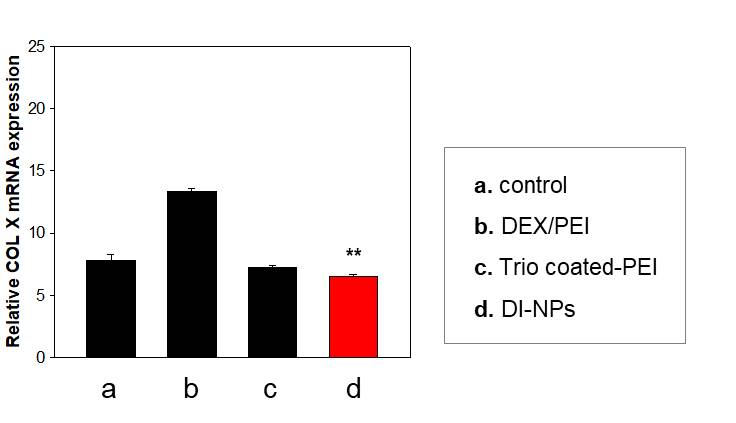
**

**Figure S7.** Evaluation of collagen type X expression from hMSCs, which were transfected with nothing (a), DEX/PEI (b), trio coated-PEI (c), and DI-NPs (d) by real-time PCR


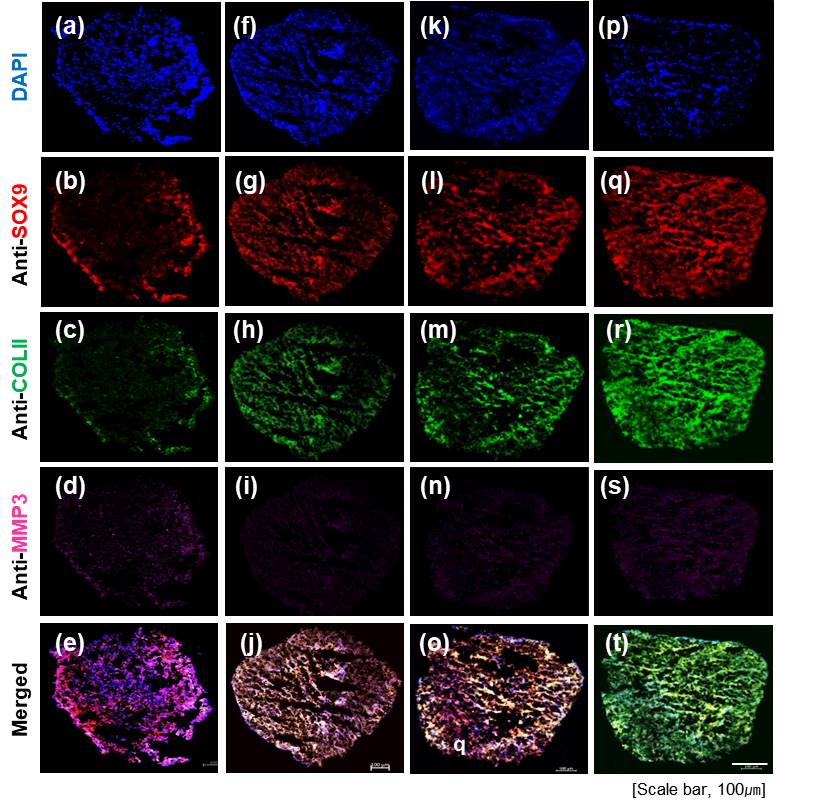


**Figure S8.** Immuno-histological analysis of chondrogenesis and osteogenesis-related markers,

including SOX9, type 2 collagen (COLII) and type 1 collagen (COLI), from hMSCs transfected

with nothing (a-e), only DP (f-j), Trio coated-PEI (k-o), DI-NPs (p-t), Scale bars, 100 µm
